# Multi-Stage Classification-Based Deep Learning for Gleason System Grading Using Histopathological Images

**DOI:** 10.3390/cancers14235897

**Published:** 2022-11-29

**Authors:** Kamal Hammouda, Fahmi Khalifa, Norah Saleh Alghamdi, Hanan Darwish, Ayman El-Baz

**Affiliations:** 1 BioImaging Laboratory, Bioengineering Department, University of Louisville, Louisville, KY 40292, USA; 2 Mathematics Department, Faculty of Science, Mansoura University, Mansoura 35516, Egypt; 3 Department of Electrical and Computer Engineering, Morgan State University, Baltimore, MD 21251, USA; 4 Department of Computer Sciences, College of Computer and Information Sciences, Princess Nourah Bint Abdulrahman University, Riyadh 11564, Saudi Arabia

**Keywords:** deep learning, classification, gleason system, grade groups, prostate cancer

## Abstract

**Simple Summary:**

Prostate cancer (PC) is the most common cancer and the second that causes death in the US. The means of PC treatment have improved with the low-risk disease for men. The Gleason system grading that comprises the Gleason score and Gleason pattern is the primary measurement to assess PC using pathological data, as well as grade groups. We have developed an automated diagnostic deep learning system for the Gleason system, which significantly affects the final treatment and is considered a valuable decision-support tool for PC patients.

**Abstract:**

In this work, we introduced an automated diagnostic system for Gleason system grading and grade groups (GG) classification using whole slide images (WSIs) of digitized prostate biopsy specimens (PBSs). Our system first classifies the Gleason pattern (GP) from PBSs and then identifies the Gleason score (GS) and GG. We developed a comprehensive DL-based approach to develop a grading pipeline system for the digitized PBSs and consider GP as a classification problem (not segmentation) compared to current research studies (deals with as a segmentation problem). A multilevel binary classification was implemented to enhance the segmentation accuracy for GP. Also, we created three levels of analysis (pyramidal levels) to extract different types of features. Each level has four shallow binary CNN to classify five GP labels. A majority fusion is applied for each pixel that has a total of 39 labeled images to create the final output for GP. The proposed framework is trained, validated, and tested on 3080 WSIs of PBS. The overall diagnostic accuracy for each CNN is evaluated using several metrics: precision (PR), recall (RE), and accuracy, which are documented by the confusion matrices.The results proved our system’s potential for classifying all five GP and, thus, GG. The overall accuracy for the GG is evaluated using two metrics, PR and RE. The grade GG results are between 50% to 92% for RE and 50% to 92% for PR. Also, a comparison between our CNN architecture and the standard CNN (ResNet50) highlights our system’s advantage. Finally, our deep-learning system achieved an agreement with the consensus grade groups.

## 1. Introduction

The American Cancer Society released the estimated cancer facts and figures for 2021. Accounting for 248,530 (26%) cases and 34,130 (11%) deaths, prostate cancer (PC) is the most common cancer and the second that causes death in the US [1], thus PC is a significant cause of morbidity and mortality. The pathological assessment of prostate biopsies determines the optimal treatment method for PC [2]. The accurate diagnosis of the whole slide images (WSIs) of the digitized prostate biopsy specimens (PBSs) assists the physician in making fundamental treatment decisions, and the early detection at the early stage for the PC leads to 100% recovery [3,4]. Different pathologists diagnosing the same sample have a 30% to 50% discrepancy in their grade groups (GG) diagnosis [5,6,7].

**Figure 1:**
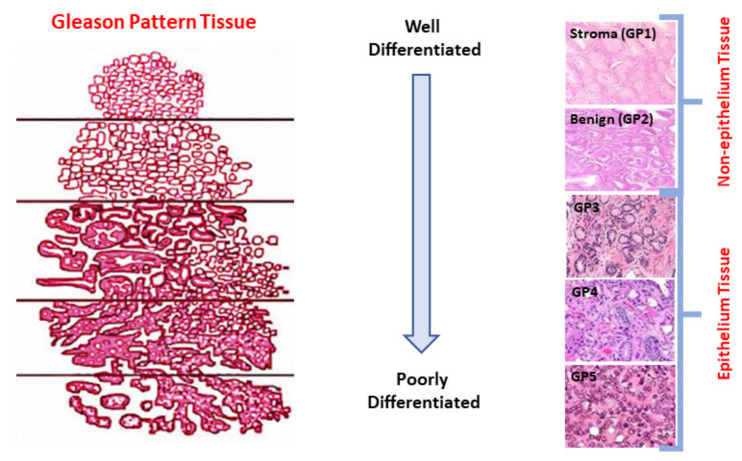
Types of Gleason Pattern (GP), which GP goes from GP1 (stroma) to GP5 depending on the cell differentiated.

The Gleason system grading (GSG) is the primary measurement to assess PC using pathological data. The GSG comprises the Gleason score (GS) and Gleason pattern (GP) that characterizes the heterogeneous tumor growth patterns observed in a biopsy regarding the discrimination degree of these tumors. Practically, the GP goes from GP1 to GP5. While the non-epithelium tissue is represented by the GP1 (stroma) and GP2 (benign), the epithelium tissue is characterized by GP3, GP4, and GP5 [5,8], see Figure 1. The GS, the grading system used to determine the aggressiveness of PC based on the two most common GP seen in the biopsy, is the primary factor in determining the stage of PC [9]. The GS varies within a typical range between 6 and 10, with 6 denoting low-grade cancer with a slow growth rate, and 10 denoting high-grade cancer that is projected to have a rapid spread.

In a relatively coarse classification, the grading system of the GS is usually categorized into just three groups: 6, 7, and 8:10 [9]. For example, GS7 might imply that the majority of cells are GP3, then GP4, or that the majority of cells are GP4, then GP3; however, the second situation leads to a far worse prognosis. Similarly, despite being grouped together, GS8 has a better prognosis than GS9 or GS10. Eventually, the 2014 International Society of Urological Pathology (ISUP) devised a simple PC grading system called GG, which was based on visually assessing cell differentiation and predominance of GP [10]. The GG scale runs from GG1 through GG5, with lower GG indicating less clinical risk. The relationship between the GSG (GP and GS) and GG is shown in Table 1.

### Related Work

The manual application of the GSG by a pathologist is subjective and time-consuming. Therefore, using medical imaging analysis and developing an automated deep learning (DL) system with the ability to assess PBSs with expert-level performance could be beneficial in this problem. Also, it could clinically increase the utility of prostate biopsy due to the availability of expert subspecialists. Image segmentation and classification have relied on deep learning in recent years, especially in medical images. Most researchers have developed algorithms to identify GSG using neural networks such as [8,11]. In particular, deep learning, especially the convolution neural network (CNN) and fully convolutional neural network (FCN), play an essential role in medical image analysis, and it has been proven crucial for it in many publications like [12,13,14,15,16,17,18]. The input image is fed to the network for training and identifying the interest structure. The probability maps are produced to identify one label for each pixel, with which the highest probability value is determining the pixel label.

Researchers produced many scientific works on GSG methods [8,11,19,20,21] that depend on variants of U-net. The U-net [22] is the standard and the most famous deep network for medical image segmentation from both radiology and histopathological image. It is the essential part of the algorithm or the main factor in enhancing the experimental results. For example, Silva-Rodríguez et al. [19] developed a computer-aided diagnosis systems (CAD) system to detect and segment the prostate gland in histology images using multi-resolution and residual and U-net. This model is robust for identifying the benign glands and achieves higher accuracy compared with traditional algorithms such as [23]. However, they failed to find the glands whenever they invaded the stroma.

For the complex methods with high accuracy, Avinash et al. [24] designed their Carcino-net architecture to include a pyramid pooling module that employs different convolutional kernels, thus discovering features at different scales. They show their algorithm’s high accuracy on low-resolution images that include large cancer areas. Arvaniti et al. [25] incorporated a DL-based technique to develop an automated prostate cancer tissue microarray Gleason grading with staining of Hematoxylin and Eosin (H&E). They introduced a DL system (DLS) that consists of two stages, CNN and a support vector machine (SVM). The CNN classified image patches within each biopsy and was followed by SVM, which uses features extracted from the resulting heatmap to classify the biopsy’s overall GG. Moreover, Bulten et al. [21] used DL to automate a grading system for prostate biopsies. The cycle consistent generative adversarial network (CycleGAN) technique is utilized first to make a transformation of stain easier on the external dataset of tissue microarrays. After that, a DLS, namely U-net, was trained on randomly sampled patches extracted from the training set to segment the GP. Nagpal et al. [8] also improved GS for prostatectomy whole-slide images using a DL-based technique. The authors used a total of 112 million image patches (pathologically annotated) from 1226 slides and validated them on a 331-slide independent dataset.

In this research, we incorporate DL to develop a grading pipeline system for the digitized PBSs and consider GP as a problem of classification, not segmentation as in current research studies [8,21,25]. We developed a multi-stage DL approach to reach our goal. As far as we know, our idea is the first of its kind to be applied. The GS and GG are finally determined using labels of GP with a high performance which can be compared to subspecialists with high expertise.

## 2. Methods

**Figure 2:**
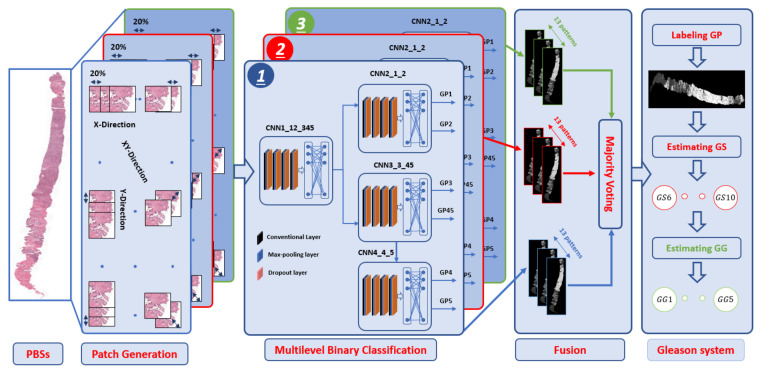
The proposed pipeline: PBSs is the digitized prostate biopsy specimens, GP is the Gleason pattern, GS is the Gleason score, and GG is the grade group.

Our target is to develop an automated DL system that performs the GSG (GP and GS) and identifies the GG. The pipeline consists of three steps, preprocessing with patch generation, multilevel binary classification, and estimation of the GSG with GG, see Figure 2.

### 2.1. Dataset Description

We used 3080 WSIs of the digitized PBSs, 2888 for training, 96 for validation, and 77 for testing. The Radboud University Medical Center, USA, supplied us with the dataset, and the University of Louisville Hospital analyzed it [21,26]. The pathologists used a semi-automatic labeling technique to circumvent complete manual annotation. They identified the GSG (GP and GS) and GG for all slides. We used Python and Matlab to implement the software using a Dell Precision workstation with 128 GB RAM and an Intel Xeon eight-core CPU running at 3.31 GHz.

### 2.2. Preprocessing Step

**Figure 3:**
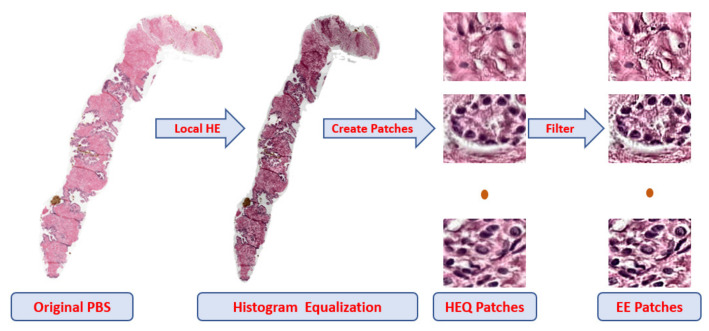
Preprocessing steps: PBSs are the digitized prostate biopsy specimens, HEQ is histogram equalization, and EE is edge enhancement.

For the trained model, the preprocessing for the input data of the CNN starts with histogram equalization (HEQ) for the PBSs to adjust intensity values and make all signals in the same range [27]. Figure 3 presents the effect of applying HEQ on the digitized PBSs. We split the HEQ images into three levels with various sizes of patches 100 × 100, 75 × 75, and 50 × 50 pixels. Only during the model training, The background ratio is a factor for selecting a patch. We choose the patch that has more than fifty percent tissue. Algorithm 1 shows the successive steps for the preprocessing. After that, the edge enhancement (EE) [28,29] is applied for the selected patches to highlight the discriminative features, see Figure 3.

For the tested model, the preprocessing step is the same as the trained model but without applying Algorithm 1 for selecting patches. We used all generated patches from the pathological tested images for the model test. Each PBS is divided into patches according to the corresponding level input image size. For the first level, the PBS is split into patches with a size of 100 × 100, which is denoted as the original patch group. After that, we generate new overlapping patches with a 20% shift for the same patch size from one direction that is the second group. Five shift steps are required to recover the original 100 × 100 patches, and thus there are four distinct generated groups of patches. Because there are three directions, X, Y, and diagonal, we generate 12 group patches plus the original one for each level of the CNN. We used three levels in our framework, with a sequential reduction in size (100 × 100, 75 × 75, and 50 × 50). Therefore, the total number of patch groups is 39.
**Algorithm 1:** Preprocessing step and selecting training patches, the value of S is 100, 75, or 50.**Input:** Whole slide images (WSIs) of the digitized prostate biopsy specimens (PBSs).**Output:** Label the choice patches into the Gleason pattern (GP) classes.Get the histogram equalization.Divide the PBSs into patches, with size S × S pixels.Select convenient training patch
Estimate the majority voting for each class in the patch (MV)Calculate two variables for corresponding patch, PC ←Patch Center and BR *leftarrow* Background ratioIf (MV==PC)&(BR≤0.5)       choose the patchElse       Remove the patch

### 2.3. Multilevel Binary Classification

**Figure 4:**
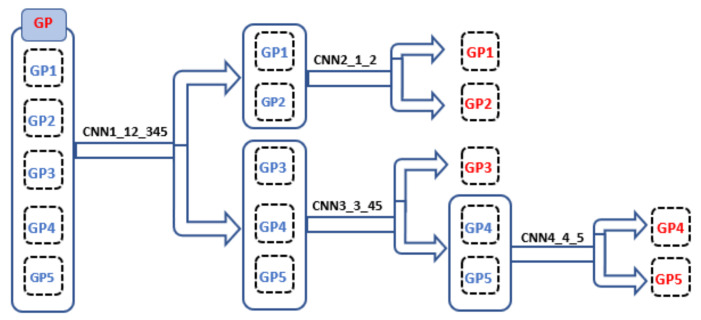
Schematic illustration of the multi-stage classification for the Gleason pattern.

The GP grade of five labels is 1 to 5 because creating CNN to classify all the five classes at once gives low classification accuracy [11]. Then, we created a multilevel binary classification to enhance the segmentation accuracy for GP. The Figure 4 shows the hierarchy of four CNNs for each level. The Gleason challenge is a complex problem for segmentation and classification techniques because the visual appearance for the malignant tissue GP 3, 4, and 5 are almost the same [5,6,7]. Therefore, we developed three levels of analysis that supply the model with different features. The small patches give the model more local information, and the large patches provide it with global details. Figure 2 depicts the proposed DL pipeline, in which the multilevel binary classification consists of four CNN in each level. Namely, we make the multilevel binary classification from scratch that consists of four CNN to identify the five labels; see Figure 2. The four CNN are CNN1 to classify group 1 (labels 1 and 2) and group 2 (labels 3, 4, and 5), CNN2 to distinguish label 1 from label 2 following CNN1. Then CNN3 separates label 3 from labels 4 and 5 combined. Finally, CNN4 separates label 4 and label 5 following CNN3.

The four CNN have the same structure at each level, but they have their own tuned parameters and size. The image is convolved with kernels in the convolution layers (CL) to extract the distinguishing features. Those features are featured maps used to identify the input patch. The kernels with size 3 × 3 are utilized in our implementation. The dimensions for the patch are reduced by two for the max-pooling layers (MPLs), decreasing the computational cost for the training time and keeping only the most prominent features. The filters are for the CLs, and the weight for the dropout layers is used to minimize CNN overfitting. The FCLs contain two layers, and the loss function is cross-entropy, as shown in Figure 3. We trained each CNN individual until reached the minimum of the cross-entropy loss. The cross-entropy loss can be defined using the following equation [30]:

(1) $$LCE=-\sum_{i=1}^{M}Y_{o}{,}_{i}log\left(P_{o}{,}_{i}\right)$$

where the number of classes is M, the binary indicator (o or 1) is $Y_{o}{,}_{i}$, and the predicted probability is $P_{o}{,}_{i}$. Tuning or hyperparameter optimization is the subset of machine learning that is the technique for selecting a set of optimal hyperparameters for a learning algorithm [31]. Many approaches are used for optimal hyperparameters, such as random search (RS), which are utilized in our models. RS approach replaces the exhaustive enumeration of all combinations by choosing them randomly. We used it in our proposed pipeline because it outperforms grid search when only a small number of hyperparameters affects the final performance of the machine learning algorithm [32]. RS supports enhancing the model’s performance and decreasing the potential for overfitting.

### 2.4. Estimating the Gleason System

The multilevel binary classification is utilized to classify the GP classes from the original PBS. The preprocessing step and patch generation give four groups for each level of PBS for three directions. The four CNN for each level is applied independently during the test. Each patch has a label according to the probability of the five GP classes relying on the binary classification of the four CNN. Therefore, each pixel has thirteen labels at any position of the image. For the same PBS with three levels, each pixel has 39 labels, see Figure 2. These 39 labels for each pixel are equivalent to the output of the 39 PBS. The pixel-wise majority voting technique for these 39 images is applied to get a single label for each pixel.

The GSG and GG have been the standard for PC diagnostic in recent years; thus, identifying them is the target for our work. Unlike the GP and GS, the WSIs of the digitized PBSs have only one label. After assigning the GP, we first identify the GS from the GP and then classify the GG. The relation between three grading GP, GS, and GG are illustrated in Table 1.

## 3. Results

We used WSIs of the digitized PBSs, for training, validation, and testing. The patches, generated for training during preprocessing with patch size at the three levels, 100 × 100, 75 × 75, and 50 × 50 pixels, number almost 4.5, 6.8, and 8.2 million, respectively. Before generating the patches, we split the digitized PBSs into train, validation, and test contorts, meaning no part of any image appears in other categories. The big challenge during the model training is the unbalanced data size, i.e., the number of patches in each class is not equal. The GP1 has a high frequency among all GP labels representing around 40%, while the GP5 has a low occurrence. Then, our trained patches contain a different number of patches for each label, and it is difficult to train our model with this strongly unbalanced data. Therefore, we defined the last label that gives patches to be GP5 and randomly selected the same number from the other four labels. For example, we used almost 35 thousand patches for each GP label at the level 100 × 100.

**Figure 5:**
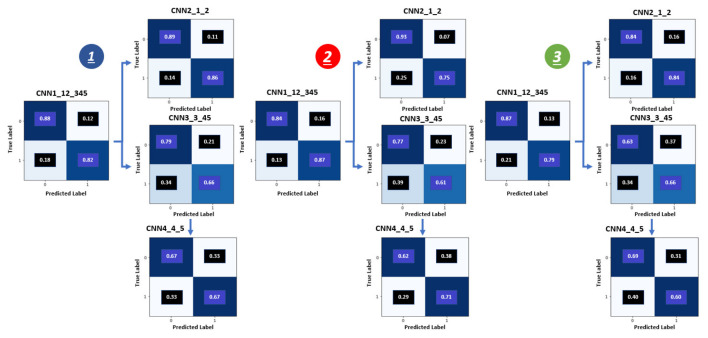
Confusion metrics for grade groups obtained by the proposed system: 1is the first level (100 × 100), 2 is the second level (75 × 75) and the third level (50 × 50).

**The multilevel binary classification** accuracy for GP is vital results for our pipeline because the accuracy of the GSG and GG depend on it. We used three metrics to evaluate the binary diagnostic of the GP classification recall (RE), precision (PR), and accuracy. The confusion matrices in Figure 5 reflect the accuracy results for these three metrics and show the relationship between the predicted and actual label for the GG results. The confusion matrices present the accuracy for the three levels (1, 2, and 3) and show the four CNN (CNN1, CNN2, CNN3, and CNN4) results inside each level.

The accuracy of the binary classification for the three-level is evaluated utilizing the performing metrics recall, precision, F1-score, accuracy [33,34] that show in the Tables see Table 2, Table 3 and Table 4. The recall, precision, and F1-score are calculated using the following equation:

(2) $$Recall=\frac{TruePositives}{\left(TruePositives+FalseNegatives\right)}$$

(3) $$Recall=\frac{TruePositives}{\left(TruePositives+FalsePositives\right)}$$

(4) $$F1-score=\frac{2\times (precision\times recall)}{\left(precision+recall\right)}$$

The overall accuracy is the proportion of correctly classified samples out of all the samples. The performance for cross classes is summarized using two metrics macro-averaged and macro-averaged. The macro-average is also estimated for each metric and calculated as simple arithmetic means of the per-class metric. The weighted average is the weighted average of each class by the number of samples from that class.

**Figure 6:**
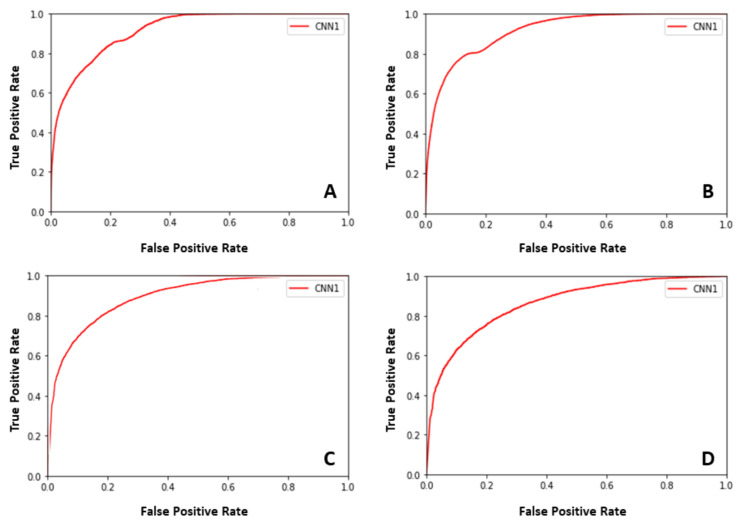
The receiver operating characteristic (ROC) curves for the proposed framework of the first level (100 × 100). CNN1 (**A**), CNN2 (**B**), CNN3 (**C**), and CNN4 (**D**).

Each level for the multilevel binary classification consists of four CNN: CNN1, CNN2, CNN3, and CNN4. The binary classification accuracy of the first level (100 × 100) for these four CNN is 0.86, 0.89, 0.75, and 0.69, respectively, see Table 2. For the second level (75 × 75), the binary classification accuracy for these four CNN is 0.82, 0.89, 0.71, and 0.65, respectively, see Table 2. For the last level (50 × 50), the binary classification accuracy for these four CNN is 0.81, 0.85, 0.68, and 0.62, respectively, see Table 3. The classification accuracy for the first level is the best among the three levels. The classification accuracy for the second level is higher than that of the third level. Increasing the size leads to enhancing the classification accuracy for the patch classification. However, it is not a generalized rule, and until the specific size, the accuracy does not improve. For our technique, increasing the size of patches to more than 100 × 100, 75 × 75, and 50 × 50 pixels does not enhance the GP labeling accuracy because a patch is assigned a GP label for all pixels inside this patch, which may have one or more GP labels in the ground truth. Figure 6 illustrates the receiver operating characteristics (ROC) curves for the proposed pipeline of the first level (100 × 100). The ROC has also been employed to support and confirm our system’s accuracy and robustness.

**Figure 7:**
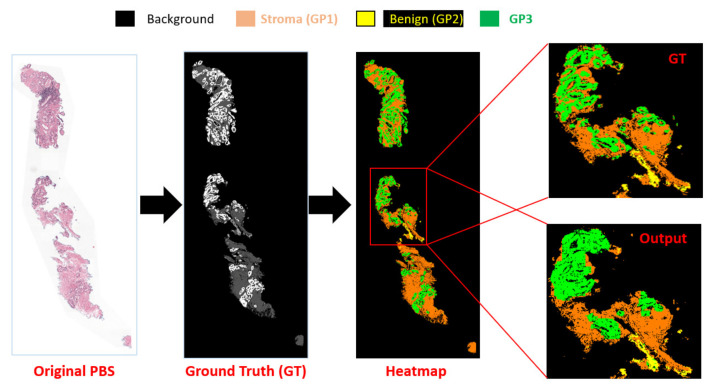
Examples of GP results from our deep learning system.

**Figure 8:**
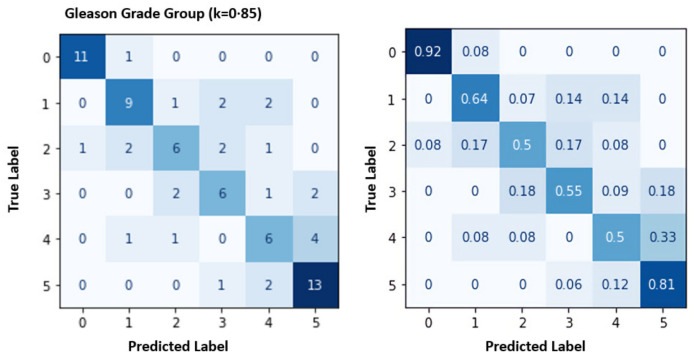
Confusion metrics for the grade groups results: cases number (**left**) and the percentage (**right**).

The target of our pipeline is to identify the GSG and GG. After classifying the patch and assigning a label for each pixel inside the PBSs, identify the GP label. The GP examples from our DL system results are shown in Figure 7 that compare the GP ground truth with the prediction after fusion of the 39 groups. Table 1 presents the fundamental converting between the GSG (GP, GS) and GG. The overall accuracy for the GG is given in Table 5 that are evaluated using two metrics, PR and RE. The grade GG results are between 50% to 92% for RE and 50% to 92% for PR. The confusion matrices in Figure 8 show the relation between the predicted and actual label for the GG results.

To highlight the value of our DL system results, we compared it with the current work by Bulten et al. [21] using the same dataset and the estimation for the various pathologists. First, Table 5 presents the comparison between our model and the Bulten et al. [21]. The performance of our model are better according to PR and RE. This comparison shows that our results have almost five percentage points advantage over current work when compared by average accuracy. Second, diagnosing according to GG is not a straightforward problem because the discordance between various pathologists is 30% to 50% [5,6,7]. The diagnostic results for our DL system are acceptable compared with pathologists’ estimated results because The GG results are between 50% to 75% for RE and 50% to 69% for PR.

The binary classification results for our created CNN, the first level, are compared against the standard CNN ResNet50 [35] to validate accuracy of our results. We generate patches using the train, validate and test data with size 224 × 224 to train and test the ResNet50. The binary accuracy for ResNet50 equivalent to the four CNN is 0.81, 0.81, 0.71, and 0.69, respectively, see Table 6. The binary classification accuracy of the first level (100 × 100) for these four CNN is 0.86, 0.89, 0.75, and 0.69, respectively, see Table 2. Therefore, our custom CNN is from 0 to 8 (mean 5) percentage points more accurate than ResNet50.

Although the binary classification accuracy between our created CNN and ResNet50 is very close, besides, our model accuracy is high. The computation cost for our deep learning is shallow compared with standard CNN because the number of parameters for our CNN is around one million. In contrast, the number of parameters for ResNet50 is more than 30 million. Also, there is another reason for creating our CNN: standard CNNs have a fixed input size. Therefore, we created three CNN of different sizes. Most of them fit with the image of size 224 × 224 pixels, but the CNN must be flexible in developing our multilevel binary classification approach.

## 4. Discussion

In the last two decades, the means of prostate cancer treatment have improved with the low-risk disease for men. As confirmed by various trials and verified using [36], radical prostatectomy is more dangerous than active surveillance. The standard gold for decision-marker is the GP and GG according to the guideline of the American Society of Clinical Oncology [37]. One of the big disadvantages for the GSG and GG is the inter-observer variability, so, enhancement of the consistency and quality of care is necessary for the prostate histopathological data prostate [37,38]. Therefore, we have developed an automated diagnostic DLS for the Gleason system, which gives significant effects on the final treatment and is considered a valuable decision support tool for prostate cancer patients. Our deep learning was developed to identify the Gleason system grading (GSG) that contains the Gleason score (GS) and Gleason pattern (GP) as well as grade groups (GG). The GS and GG grade is used to classify the malignant tissue generated only from GP 3, 4, and 5.

The architecture system was implemented for identifying the GSG of digitized PBS. Various experienced pathologists and urologic subspecialists have created ground truth for the data sets. Each one has a diverse background and at least twenty-five years of experience. For every specimen, they accessed immunohistochemically stained sections and many histologic sections. According to our experimental results, the overall diagnostic accuracy for our DLS illustrated more rate than general pathologists. Many publications such as [39,40] confirm DLS is a great diagnostic tool for two reasons. First, alert pathologists on what might be missed and identify small tissue regions. Those regions based on the judgment of the pathologist then lead to overrule false-positive categorizations. Therefore, this DLS boosts the treatment modalities chosen.

Our ultimate goal is to implement a framework with good accuracy using multi-stage classification-based DLS for the Gleason system. The diagnostic accuracy results for the proposed pipeline using multi-stage classification show that the accuracy for the three levels has very, very closed. Still, the first level, 100 × 100, has higher accuracy than the other two levels; see Table 2, Table 3 and Table 4. The classification accuracy for the first level is the best among the three levels. Besides, the classification accuracy for the second level is higher than that of the third level. Increasing the size leads to enhancing the classification accuracy for the patch classification. The accuracy for our proposed framework, the first level, is higher than the standard CNN ResNet50 [35] that presents in Table 6. This comparison reflects that our CNNs were created with high performance to fit this problem and confirm the benefits of utilizing the RS technique for hyper-parameter tuning.

The binary classification results for our created CNN, the first level, are compared against the standard CNN ResNet50 as it is one state-of-the-art method to validate the accuracy of our results. Also, we compared our approach with current research that uses the same data set. An important aspect of our work is in the structure of our DL system, i.e., the pyramidal architecture that is a multi-level pipeline with three CNNs. Despite the fact that there is the recent advantage of Vision transformer in medical image applications separating all five groups at once will be a challenging task for any Dl-based workflow due to the similarity between malignant groups. Therefore, our pyramidal architecture allowed the designed system to extract various information to help in such a task (all at once classification of five classes). For example, the larger patches give more global information, while the small patches provide local details. We cannot use standard CNN like ResNet50 instead of our created CNNs because all standard CNN are fixed size, i.e., 224 × 224, and our approach utilizes CNN of dynamic size.

Our proposed DLS for diagnosing GSG is useful in healthcare systems; like enhancing grading consistency, decreasing the morbidity and mortality of low-risk diseases, and reducing consultation-associated costs. Classification accuracy for identifying the GG using the performance metrics precision and recall, the G1 grade has the highest accuracy. The G2 grade has a high accuracy compared with G3, G4, and G5 grades, and all are less than G1. Therefore, our proposed system could be perfect for classifying low-risk cases accurately. Then, it is qualified for more conservative management.

The most famous technique for classifying PC is GG which is the main system for determining the PC treatment [36,41]. Though the GG classification is not a straightforward task, there is no match between general pathologists’ and subspecialists’ results. The general pathologists’ grading is less concurrent than the subspecialists’ grading [42]. However, there is discordance between subspecialists because of the difficulty of GG and inherent subjectivity. Overcoming these disagreements is essential to improving PC risk stratification. We prioritize developing a system for predicting clinical risk and identifying grades with high precision. Artificial intelligence models, especially DL, can differentiate novel histoprognostic signals. This is essential for assistance in stratifying patient risk like existing molecular tests [43] and helping for specimens that the human eye cannot discover [44,45].

Although our proposed DLs have promising results, there are some limitations. First, the data sets were collected from only institutions used to train and test our proposed pipeline. To improve the robustness of our system, we should test the model using external data set from another institution that helps to test several staining protocols for WSI. Second, each biopsy was labeled and treated independently by pathologists. Many biopsies are sampled from many regions of the prostate for clinical practice. Therefore, various biopsies can be used after updating our model and providing a grade group prediction at the patient level. Finally, our work concentrated on grading acinar adenocarcinoma in prostate biopsies. However, there are other types of tumors that can identified in prostate biopsies, like colon glands. Also, it could contain more prognostic information like intraductal carcinoma detection [46].

## 5. Conclusions

We developed a DL system based on multilevel binary classifications to identify the Gleason system grading (GSG) and grade groups (GG), and it was run on the WSIs of the PBSs. The core for our system is the multilevel binary classifications, and we utilized it to identify the GP, which includes three levels of different sizes. We created from scratch four CNN that run 39 groups for each PBS. Finally, we used the GP label to classify the Gleason score (GS) and Grade Groups (GG). We will test this model in the blind dataset to make it robust and useful in assistive clinical tools.

In future work, because the digitized PBSs do not have the same orientation, adding a new preprocessing step to overcome this challenge will fit our results. Namely, a data augmentation to rotate the overlapped patches with angles 45 and 90, as well as flipping them, will enhance our pyramidal CNN accuracy. In addition, to highlight our model, we will try to test our model in other datasets from another institution, comparing our pipeline with other deep networks from other research work, and using more than one optimal hyperparameter to train our deep network to help to enhance accuracy and selection.

## Figures and Tables

**Table 1:** The relation between the Gleason pattern (GP), Gleason score (GS), and grade groups (GG) system.

GP	Risk Level	GS	GG
GP1	Stroma	-	-
GP2	Benign	-	-
GP3	Low	GP3 + GP3 = GS6	GG1
	Favorable	GP3 + GP4 = GS7	GG2
GP4	Unfavorable	GP4 + GP3 = GS7	GG3
	High	GP4 + GP4 = GS8 GP3 + GP5 = GS8 GP5 + GP3 = GS8	GG4
GP5	High	GP4 + GP5 = GS9 GP5 + GP4 = GS9 GP5 + GP5 = GS10	GG5

**Table 2:** Classification accuracy for the first level (100 × 100) of our proposed model, containing four CNNs.

	CNN1_12_345			CNN2_1_2			CNN3_3_45			CNN4_4_5		
	Precision	Recall	F1-Score	Precision	Recall	F1-Score	Precision	Recall	F1-Score	Precision	Recall	F1-Score
**Class1**	0.89	0.88	0.88	0.97	0.89	0.93	0.58	0.79	0.67	0.67	0.66	0.66
**Class2**	0.81	0.82	0.82	0.66	0.86	0.75	0.83	0.66	0.74	0.66	0.67	0.67
**Performance Across Classes**												
**Macro-averaged**	0.85	0.85	0.85	0.81	0.88	0.84	0.71	0.72	0.70	0.66	0.66	0.66
**Weighted-average**	0.86	0.86	0.86	0.91	0.89	0.89	0.74	0.71	0.71	0.66	0.66	0.66

**Table 3:** Classification accuracy for the second level (75 × 75) of our proposed model, containing four CNNs.

	CNN1_12_345			CNN2_1_2			CNN3_3_45			CNN4_4_5		
	Precision	Recall	F1-Score	Precision	Recall	F1-Score	Precision	Recall	F1-Score	Precision	Recall	F1-Score
**Class1**	0.97	0.84	0.90	0.79	0.93	0.86	0.55	0.77	0.64	0.68	0.62	0.65
**Class2**	0.54	0.87	0.66	0.92	0.75	0.83	0.81	0.61	0.70	0.65	0.71	0.68
**Performance Across Classes**												
**Macro-averaged**	0.75	0.85	0.78	0.85	0.84	0.84	0.68	0.69	0.67	0.66	0.66	0.66
**Weighted-average**	0.89	0.85	0.86	0.85	0.84	0.84	0.71	0.67	0.67	0.66	0.66	0.66

**Table 4:** Classification accuracy for the third level (50 × 50) of our proposed model, containing four CNNs.

	CNN1_12_345			CNN2_1_2			CNN3_3_45			CNN4_4_5		
	Precision	Recall	F1-Score	Precision	Recall	F1-Score	Precision	Recall	F1-Score	Precision	Recall	F1-Score
**Class1**	0.89	0.87	0.88	0.84	0.84	0.84	0.53	0.63	0.57	0.63	0.69	0.66
**Class2**	0.76	0.79	0.78	0.84	0.84	0.84	0.75	0.66	0.70	0.66	0.60	0.63
**Performance Across Classes**												
**Macro-averaged**	0.83	0.83	0.83	0.84	0.84	0.84	0.64	0.64	0.64	0.64	0.64	0.64
**Weighted-average**	0.85	0.85	0.85	0.84	0.84	0.84	0.66	0.65	0.65	0.64	0.64	0.64

**Table 5:** The comparison between our DL approach and the current work for grade groups results.

	Our Model		Bulten et al. [21]	
	Precision	Recall	Precision	Recall
**Benign**	0.92	0.92	0.95	0.94
**GG1**	0.64	0.69	0.65	0.70
**GG2**	0.50	0.60	0.44	0.51
**GG3**	0.55	0.55	0.40	0.52
**GG4**	0.50	0.50	0.34	0.33
**GG5**	0.81	0.68	0.89	0.67

**Table 6:** Classification accuracy for the ResNet50 with the patch size 224 × 224.

	CNN1_12_345			CNN2_1_2			CNN3_3_45			CNN4_4_5		
	Precision	Recall	F1-Score	Precision	Recall	F1-Score	Precision	Recall	F1-Score	Precision	Recall	F1-Score
**Class1**	0.68	0.79	0.73	0.81	0.82	0.82	0.59	0.92	0.72	0.53	0.63	0.75
**Class2**	0.88	0.82	0.85	0.82	0.80	0.81	0.91	0.57	0.70	0.80	0.72	0.75
**Performance Across Classes**												
**Macro-averaged**	0.78	0.80	0.79	0.81	0.81	0.81	0.75	0.74	0.71	0.66	0.67	0.67
**Weighted-average**	0.82	0.81	0.81	0.81	0.81	0.81	0.78	0.71	0.71	0.71	0.69	0.69

## Data Availability

Materials, data, and associated protocols will be available to readers after the manuscript being accepted.
